# Does local anaesthetic reduce pain in rubber ring castration of neonatal lambs?

**DOI:** 10.18849/ve.v9i1.658

**Published:** 2024-01-10

**Authors:** Hannah Higgins+

**Keywords:** ANALGESIA, CASTRATION, LAMBS, LOCAL ANAESTHESIA, LOCAL ANAESTHETIC, PAIN, RUBBER RINGS

## Abstract

**PICO question:**

In lambs less than 7 days old undergoing castration with rubber rings does administration of local anaesthetic compared to no local anaesthetic result in a reduction of pain-related behaviours?

**Clinical bottom line:**

**Category of research:**

Treatment.

**Number and type of study designs reviewed:**

Six studies were appraised; all were controlled clinical or field trials.

**Strength of evidence:**

Moderate.

**Outcomes reported:**

Local anaesthetic administered to lambs castrated with rubber rings resulted in the demonstration of fewer pain related behaviours and also diminished the increases in plasma cortisol in the immediate post-castration period when compared to lambs castrated without local anaesthetic. Local anaesthetic administered at least 15 minutes before rubber ring castration may significantly reduce behavioural signs of pain and plasma cortisol changes.

**Conclusion:**

In lambs less than 7 days old undergoing castration with rubber rings, local anaesthetic reduces markers of pain when compared to lambs castrated without local anaesthetic.

## How to apply this evidence in practice

The application of evidence into practice should take into account multiple factors, not limited to: individual clinical expertise, patient’s circumstances and owners’ values, country, location or clinic where you work, the individual case in front of you, the availability of therapies and resources.

Knowledge Summaries are a resource to help reinforce or inform decision-making. They do not override the responsibility or judgement of the practitioner to do what is best for the animal in their care.

## Clinical scenario

A commercial sheep farmer asks you about the use of local anaesthetic (LA) for his male lambs which are castrated at 24 hours old using rubber rings. Legislation in the UK does not require anaesthetic for this method of castration in lambs less than 7 days old; the supermarket he sells his lambs to are encouraging LA at routine castration and have suggested a device called Numnuts® could be used. The farmer thinks that the pain experienced by lambs is insignificant to justify the extra time and effort involved in injecting LA before applying rubber rings. You search for evidence to inform your advice.

## The evidence

There is moderate evidence to suggest that using local anaesthetic in lambs less than 7 days old at the time of castration with rubber rings provides pain relief, and that administration of local anaesthetic 15 minutes before castration gives superior pain relief to administration at the same time as castration. Overall the findings of most of the papers reviewed are consistent. All of the cited papers are controlled clinical or field trials and the majority are randomised, therefore they are high on the hierarchy of evidence.

## Summary of the evidence

**Kent et al. (1998):** 

Population	Suffolk x Greyface lambs aged 4–8 days. The location is not reported.
Sample Size	Not stated but calculated to be 120 lambs.
Intervention Details	Fifteen groups of eight lambs. Seven groups were involved in a study focusing on tail docking only (this does not relate to the PICO question and therefore will not be commented on further in this Knowledge Summary). Eight groups were used for the castration study. Treatment groups: Handled control, no local anaesthetic (LA) (n = 8). Handled control, LA (0.2 ml of lignocaine hydrochloride 2% injected into each the left and right sides). Site and injection method not clear (n = 8). Castrated with prototype bloodless castrator and rubber ring, no LA (n = 8). Castrated with prototype bloodless castrator and rubber ring, 0.2 ml of lignocaine hydrochloride 2% injected into left and right sides of cranial aspect of the scrotum with a high-pressure needleless injector immediately before castration (n = 8). Castrated with rubber ring, no LA (n = 8). Castrated with rubber ring, 0.2 ml of lignocaine hydrochloride 2% injected into each the left and right sides of cranial aspect of the neck of the scrotum with a high-pressure needleless injector immediately after rubber ring placement (n = 8). Castrated with rubber ring, 0.2 ml of lignocaine hydrochloride 2% injected into each testis with high-pressure needleless injector immediately before rubber ring placement (n = 8). Castrated with rubber ring, 0.2 ml of lignocaine hydrochloride 2% injected into each the left and right sides of cranial aspect of the neck of the scrotum with a needle and syringe immediately after ring placement (n = 8).
Study design	Controlled trial.
Outcome studied	Subjective: behavioural responses to treatment. Lying and standing postures for 120 minutes after treatment measured as time in minutes spent in these postures. Active behavioural responses (restlessness, rolling, jumping, stamping, kicking, easing quarters, head turning and vocalisation) for 96 minutes after treatment measured as summative incidence of these behaviours (restlessness, rolling, stamping, kicking and easing quarters). Objective: plasma cortisol at 20, 40, 60, 80, 120 and 180 minutes after treatment.
Main findings (relevant to PICO question)	When compared with no anaesthetic, all methods of local anaesthetic significantly reduced, but did not eliminate entirely, the pain associated with rubber ring castration. Rubber ring castration with no LA resulted in a mean of 86 minutes (± 1) spent in abnormal postures (abnormal lying: partial or full extension of hindlegs, or lying laterally with one shoulder off the ground; abnormal walking: walking with abnormal swaying or stamping movements; statue standing: standing still). This was significantly more (P ≤ 0.01) than rubber ring castration with each of injection of LA into the testes (51 ± 22 minutes), neck of the scrotum (36 ± 14.3 minutes) and spermatic cord (36 ± 15.7 minutes). Rubber ring castration with no LA resulted in a mean of 216 incidences (± 108) of active behaviours. This was significantly more (P ≤ 0.01) than rubber ring castration with either an injection of LA into the testes (95 ± 50 incidences), neck of the scrotum (69 ± 41 incidences) or spermatic cord (77 ± 43 incidences). Rubber ring castration with no LA resulted in a mean peak plasma cortisol of 160 nmol l^–1^ (± 53.3). This was significantly more (P ≤ 0.01) than rubber ring castration with each of injection of LA into the testes (67 ± 44.7 nmol l^–1^), neck of the scrotum (80 ± 53.1 nmol l^–1^) and spermatic cord (75 ± 55.2 nmol l^–1^).
Limitations	Sample size was not explicitly stated. No explanation of how sample size was determined or of power calculations. Allocation to treatment groups was not randomised but groups were balanced for weight and age. Behavioural scoring was not blinded. It is not clear how the behavioural scoring was standardised.

**Kent et al. (2004):** 

Population	Crossbred lambs under 4 days old, from seven different farms in Scotland.
Sample Size	600 lambs.
Intervention Details	Ten shepherds each castrated and tail docked 60 lambs, 15 per day over 4 consecutive days. In each flock lambs were randomly allocated to one of four treatments. Treatment groups: 10 lambs castrated and tail docked with rubber rings. 20 lambs castrated and tail docked with rubber rings and bloodless castrator. 20 lambs given local anaesthetic (lignocaine hydrochloride 2%) and castrated and tail docked with rubber rings (control group). 0.3 ml into each testis and 0.3 ml under each rubber ring immediately after rubber ring application (four flocks). 0.3 ml under each rubber ring immediately after application (six flocks). 10 lambs placebo injection of 0.02 mg/ml lignocaine hydrochloride as above and castration and tail docking with rubber rings (placebo group). It is not stated whether the lignocaine is administered following the protocol in 3.I or 3.II.
Study design	Blinded randomised controlled field trial.
Outcome studied	Objective behavioural measurements in 14–44 minutes after treatment Lambs were observed by one of a group of nine observers trained to look for pain-related behaviours. Incidence of restlessness, rolling, jumping, foot stamping, kicking, easing quarter and tail wagging scored combined to give an ‘abnormal activity’ (restlessness, easing quarters, tail wagging (REW) score. Total time spent in postures: lying with hind limbs fully extended, lying with hind limbs partially extended, statue standing, standing with abnormal movements. Subjective pain assessment. Visual analogue scales (VAS) used to give a pain score based on a 2 minute observation period within 15–45 minutes after treatment. Long term outcomes. Time to lose scrotum. Size and score of scrotal lesions (measured on one occasion between 24 and 32 days after castration).
Main findings (relevant to PICO question)	Local anaesthetic significantly reduced the abnormal activity scores compared to the placebo and control groups. Rubber ring treatments had 115 incidences (Interquartile range (IQR) 78–161), which was significantly (P ≤ 0.05) more than those receiving LA with 25 incidences (IQR 11–54). The REW score was not significantly different between the two LA groups. Local anaesthetic significantly reduced some abnormal lying behaviours (lying with legs fully extended). Lambs treated with rubber rings and no LA spent 22 minutes (IQR 6–26) lying with hind limbs fully extended, compared to 6 minutes (IQR 0–23) for those receiving LA. Local anaesthetic significantly reduced the visual analogue pain scores compared to placebo and control groups. VAS scores were significantly (P ≤ 0.05) lower for lambs being castrated and tail docked with LA (2.1 IQR 0.8–0 and 1.9 IQR 1.2–3.2) compared to without (5.4 IQR 4.0–7.7 and 7.1 IQR 5.1–8.0) when observed by the shepherds and the observers respectively. 39% of the 75 of lambs castrated with rubber rings had lost their scrotum by 24–32 days after castration, significantly (P ≤ 0.05) fewer than the 65% of the 145 that had been castrated using LA. Scrotal lesion width and score (measured on one occasion between 24 and 32 days after castration) were also significantly lower for those castrated with LA than without. There was no significant difference in response between the lambs receiving LA under the rubber ring only, or under the rubber ring and in the testes combined.
Limitations	The treatments were performed by ten different operators; technique may have varied and influenced the pain response. The 600 lambs were not equally spread between the ten flocks therefore the number of lambs receiving each treatment varied between flocks. The behavioural measurement outcome is described as objective but there may be a degree of subjectivity. The shepherds assigning pain scores using the visual analogue scale had no training in pain behaviours and were reliant on their own experience.

**Mellema et al. (2006):** 

Population	White Swiss Mountain and White Swiss Mountain x Charolais ram lambs ages 2–7 days. Location is not reported.
Sample Size	70 lambs.
Intervention Details	Lambs were randomly allocated to one of six treatments. Treatment groups: Castration with rubber ring after injection of local anaesthetic (lidocaine hydrochloride 2% at a dose rate of 4 mg/kg bodyweight diluted with saline to 5 ml. 1.5 ml administered into each spermatic cord, 2 ml subcutaneous injection (SC) around the scrotal neck) (n = 15). Castration with rubber ring and injections as above but with 5 ml saline (no LA) (n = 10). Burdizzo castration with local anaesthetic (as for treatment group 1) (n = 15). Burdizzo castration with saline injection (as for treatment group 2) (n = 10). Control handling and local anaesthetic (as for treatment group 1) (n = 10). Control handling and saline injection (as for treatment group 2) (n = 10). All injections took place 5 minutes before castrations / handling. The syringes for injection of LA or saline were prepared by a second person so the person administering them was blind to the contents.
Study design	Blinded randomised controlled trial.
Outcome studied	Subjective measurements of overall behavioural response, struggling, vocalisation and lip curling were recorded and added together to give a cumulative ‘pain score’. Objective measurement of plasma cortisol was analysed using the mean area under the curve for the 50 observed minutes in the 120 minutes after castration. Active behaviour and postures were recorded for five 10 minute periods in the 2 hour immediately after treatment. Scrotal region assessment showed no significant difference in the first and last observed pain response between rubber ring castration with or without LA. Bodyweight measurement was not significantly affected by treatment group.
Main findings (relevant to PICO question)	For lambs castrated with rubber rings, LA significantly reduced the immediate pain response (pain score). The authors report that the use of local anaesthesia (LA) tended to reduce the pain response but this was not significant (P = 0.132); the data is not reported. Rubber ring castration without LA gave a median pain score of 1 out of 8 (range 0–4), rubber ring castration with LA gave a median pain score of 0 out of 8 (range 0–4). Lambs castrated with rubber rings and LA showed no significant change in plasma cortisol compared to the baseline whereas lambs castrated with rubber rings and not receiving LA showed significant cortisol increase. LA significantly reduced the peak cortisol level and the area under the curve after castration with rubber rings, compared to no LA. Rubber ring castration with LA (2634 ± 5450 nmol/L minute) was not significantly different from either of the control groups (P > 0.05) but was significantly lower (P > 0.05) than for rubber ring castration without LA (13324 ± 6617 nmol/L minute). The rate ratio for expression of active behaviour was more than ten times higher in the 2 hours after castration for lambs castrated with rubber ring and no LA than rubber rings with LA. LA reduced the total active behaviour scores for lambs castrated with rubber rings to the control level. Lambs castrated with rubber rings and no LA continued to show significantly more active behaviours in the 1–6 days after castration than lambs castrated with rubber rings and LA. Castration with rubber rings and no LA showed significantly more abnormal postures in the 2 hours after castration than those with LA, but beyond this the proportion of abnormal postures did not vary between groups. Active behaviours and postures rubber ring castrated lambs without LA showed a 10.8 times higher rate ratio (95% CI 7.6–15.3 rate ratio) for total active behaviour in the 50 observed minutes in the 2 hours after castration than those with LA. This was significant at P < 0.05. Those with LA showed similar active behaviours to the control groups.
Limitations	There is no power calculation for sample size. Observer was blinded to the injection types (lidocaine or saline) but could not be blinded to the castration method. Lambs were randomly allocated to treatment, but groups were not balanced for weight or litter size. There are no details of how the observer was trained or experienced in identifying the specific behaviours measured.

**Molony et al. (2012):** 

Population	Greyface x Texel lambs aged 2–3 days old in Scotland.
Sample Size	24 lambs.
Intervention Details	Three groups of eight lambs: Castration with conventional rubber rings. Castration with novel, smaller, tighter rubber rings. Castration with novel, smaller, tighter rubber rings with injection of 0.3 ml of 5% procaine by needleless injector into each spermatic cord immediately before rubber ring placement.
Study design	Randomised controlled trial.
Outcome studied	Active behavioural responses and postures for 1 hour after castration. Lesions at site of castration: Lesions were scored and the time of scrotum sloughing was recorded and were not significantly different between groups. Chronic pain: Behaviours consistent with chronic pain were recorded twice weekly for 4 weeks; no significant difference between groups was identified (P > 0.32). Daily live weight gain: Lambs weighed immediately prior to castration, at 3 days post castration and then twice weekly for 4 weeks. There was no significant difference between groups (P = 0.83).
Main findings (relevant to PICO question)	LA at castration significantly reduced time spent in most active behaviours compared with the two groups not given anaesthetic. Total time spent in activities indicating pain behaviours had a median of 279.8 minutes (interquartile range (IQ) 205.5–298 minutes) for lambs castrated with the novel rubber ring and no local anaesthestic (LA) and 286.5 minutess (IQ 218–382.6 minutes) for lambs castrated with conventional rubber rings and no LA. These were both greater than 105.8 minutes (IQ 68.8–191.6 minutes) when LA was used before rubber ring placement (P < 0.05 in each case). LA significantly reduced abnormal lying postures compared with the two groups not given LA. Total time spent in abnormal lying postures for lambs castrated with the novel rubber rings had a mean of 45.5 minutes (± 4 minutes), and 50.5 minutes (± 1.8 minutes) for conventional rubber rings, which were both greater than 26.3 minutes (± 6.9 minutes) when LA was used before novel rubber ring placement (P < 0.05 in each case). In the 4 weeks after treatment, no effect was seen on the chronic pain behaviours between any of the groups.
Limitations	The method of randomisation was not described. No details of who conducted the scoring or how it was standardised. No blinding. LA was only administered to lambs being castrated with the novel rubber rings; no direct comparison could be made between standard rubber rings with or without local anaesthetic. Small sample size, although this may be appropriate, a power calculation would help to justify.

**Thornton & Waterman-Pearson (1999):** 

Population	Crossbred lambs aged 4–6 days in England.
Sample Size	216 lambs.
Intervention Details	4x3x3 block design where each lamb was assigned to one of four groups (three castration methods and a control) and one of three anaesthetic regimes. There were 36 possible combinations and groups of six lambs received each combination. Groups: Controlled handling. Rubber ring. Rubber ring and clamp. Surgical. Anaesthetic regime: No local anaesthetic (LA). LA – 2% lignocaine: 0.5 ml into each spermatic cord, 1 ml into the skin at the site of the rubber ring, 0.5 ml into each testis. General anaesthetic – inhalational anesthesia with 2% halothane in 100% oxygen.
Study design	Factorial randomised controlled trial.
Outcome studied	Pain assessed using a visual analogue scale (VAS) measuring active pain avoidance, unresponsive behaviours to interaction and scrotal pain Mechanical nociceptive threshold response assessment. Plasma cortisol concentration increase from 15 minutes to 1 hour after castration with rubber rings. This was reduced by use of LA to levels that were not significantly different from the control. No values were given.
Main findings (relevant to PICO question)	Administration of local anaesthetic reduced the levels of all visual analogue scales, for lambs castrated with rubber rings, to similar to those for the control group. These were all reduced to near control levels by the administration of LA. The reduction in pain avoidance behaviour and scrotal pain was significant (P < 0.05) but the values are not given. Mechanical nociceptive threshold response assessment demonstrated a slight (not significantly different from control) rise in rubber ring castration which was abolished following the use of LA. Values are not given. For lambs castrated with rubber rings, those receiving local anaesthetic had significantly reduced active pain behaviours and scrotal pain compared with no anaesthetic and general anaesthetic. There was no difference in the mechanical nociceptive response between lambs castrated with rubber rings that receive local anaesthetic or not. In lambs receiving LA none of the castration methods produced changes in cortisol that were significantly different from the control.
Limitations	There was no calculation for the sample size or statistical power. A study measuring multiple interventions and outcomes such as this needs a large sample size to be confident of the significance of the results. The study was conducted over two successive lambing seasons; there was no detail any variations between each season that may have influenced results. VAS assessment was subjective. The method used for VAS has been used by other researchers, but it is unclear whether is it validated. Assessment of behavioural scores was not blinded The authors do not provide details of the data collected or consistently report P values.

**Wood et al. (1991):** 

Population	Dorset x Finnish Landrace male lambs aged 5–6 days. Location not stated.
Sample Size	36 lambs.
Intervention Details	Six groups of six lambs each subjected to one of the following treatments. Control handling without no local anaesthetic (LA). Castration and tail docking with rubber rings, LA. LA only, 15–20 mins before controlled handling. LA protocol: Caudal epidural with 0.3ml of 2% lignocaine. 5 ml 2% lignocaine into each spermatic cord. 1 ml 2% lignocaine into neck of the scrotum. 5 ml 2% lignocaine into each testis. LA protocol as for group 3 plus castration and tail docking 15–20 mins after LA. Intravenous (IV) naloxone only: 0.2 mg kg^–1^ naloxone (0.2 mg ml^–1^) by slow IV injection 10–12 mins before controlled handling and again 1 hour later. IV naloxone as for group 5 plus castration and tail docking.
Study design	Controlled clinical trial.
Outcome studied	Subjective: behavioural measurements of pain for 240 minutes after castration and tail docking. Objective: plasma cortisol for 240 minutes after castration.
Main findings (relevant to PICO question)	Pain behaviours when LA was administered before castration were not significantly different from the control (P > 0.05). Primary data values were not given. Plasma cortisol was not significantly different from the control (mean = 30.9 nmol l^–1^ ± 14.51 nmol l^–1^) when LA was administered before castration (mean = 34.8 nmol l^–1^ ± 14.51 nmol l^–1^) (P > 0.05). LA given 15–20 minutes before castration and tail docking with rubber rings suppressed behavioural and cortisol changes associated with castration and tail docking to a level in line with the lambs that were handled only (control handling group).
Limitations	This is a small sample size and there is no explanation of how sample size was determined or power calculation. Allocation to treatment groups was not randomised. The authors do not state whether the observers were blinded to the treatment groups. There is no explanation of who recorded behavioural measurements or how this was standardised. The authors infer that LA had the effect of reducing the behavioural and cortisol changes associated with castration and tail docking. However, the results are presented as bar charts, without precise values, and P values are only given for the difference in values for the control handled group and the castration and tail docking group without LA. Observations absent for 1 lamb at 2 points, which is reported in the figure legends but not accounted for in the text.

## Appraisal, application and reflection

In the UK there is currently no legal requirement for local anaesthestic (LA) to be used when castrating lambs less than one week old with rubber rings.

The papers reviewed here covered a 30 year period of research into pain reduction for castration in lambs. Markers of pain used for outcomes were mostly plasma cortisol (Mellor & Murray, 1989) and behavioural expressions and postures which have been demonstrated to be sensitive markers of pain associated with castration in lambs (Mellor & Murray, 1989; Molony et al., 1993; and Molony et al., 2002). A method of assessing acute pain in lambs has been validated by Molony et al. (2002) and these observations formed the basis for behavioural measurements in many of the papers reviewed. Power calculations for sample size were not included in any of the papers and confidence intervals were rarely reported in the results.

Wood et al. (1991) concluded that lignocaine injected into the spermatic cords, scrotal neck and testes concurrently 15–20 minutes before castration resulted in the demonstration of fewer pain related behaviours and also diminished the increases in plasma cortisol in the immediate post-treatment period compared to those castrated without LA. The lambs receiving LA showed behavioural and cortisol responses close to those of the control group. This was, however, a study of small sample size that was not randomised or blinded, giving potential for bias in the results, therefore the power of the study is also unknown.

Thornton & Waterman-Pearson (1999) used the same LA protocol as Wood et al. (1991) in their study and also found that LA reduced plasma cortisol to similar levels to the control, and significantly reduced pain behaviours demonstated by lambs when compared with castration without LA. This study was the only one to assess scrotal pain as an outcome and found than LA significantly reduced the scrotal pain associated with rubber ring castration. This was a complex factorial study and although the sample size was larger than most of the other studies, only six lambs received each combination of treatments and outcome measures.

Whilst these studies (Thornton & Waterman-Pearson, 1999; and Wood et al., 1991) suggest that LA does significantly reduce the pain associated with rubber ring castration, in both cases the LA was administered 15 minutes before rubber ring application. On commercial sheep farms it may not be practical or efficient to handle each lamb twice and allow 15 minutes to elapse between injection of LA and application of the rubber ring. The other studies reviewed here investigate the effect of LA injected immediately before or after rubber ring application.

Studies conducted in Australia and New Zealand have demonstrated that lambs receiving LA immediately before or after rubber ring application showed fewer behaviours associated with pain in the first hour after castration (Jongmanet al., 2016; Small et al., 2020; and Stewart et al., 2014). However, as it is common in these countries to castrate lambs at ‘marking’ (marking being the gathering of lambs for the procedures of tail docking, castration of males, ear marking, ear tagging, vaccination, and drenching, often performed at between 6 and 12 weeks of age), rather than in the neonatal period, all of these studies used a population of lambs over four weeks old. The testes would be more developed, with a larger scrotal neck, than lambs less than seven days old being castrated in the UK, consequently the findings may not be directly applicable to the UK population.

In lambs less than 8 days old painful behaviours and plasma cortisol are significantly reduced if LA is administered at the time of rubber ring placement (Kent et al., 1998; Kent et al., 2004*; *Mellema et al., 2006; and Molony et al., 2012). LA was injected subcutaneously into the neck of the scrotum (in at least two sites in order to create a rubber ring block), the middle of the testes, into the spermatic cords or a combination of some or all of these sites, at the time of rubber ring application. All protocols gave similar results i.e. a significant reduction in pain related behaviours but not complete elimination. One study (Mellema et al., 2006) found that the subjective assessment of immediate pain was not significantly reduced, but other behavioural measurements based on the validated method were. In addition, the plasma cortisol did not significantly change compared to the control. These studies are mostly randomised controlled trials and provide strong evidence that pain is relieved by the use of LA at the time of rubber ring application, excluding the Kent et al. (1998) study which was not randomised.

Injecting LA at the time of castration is more time-efficient than injecting it 15 minutes before but there is still a time cost; rubber ring castration takes an average of 29 seconds and injection into the scrotal neck followed immediately by rubber ring castration takes an average of 68 seconds (Kent et al., 2004). Efforts have been made to further streamline the process by combining the LA injection and rubber ring application into one action using a preparatory device, Numnuts®, which delivers 1.5 ml lignocaine 2% into the dorsal midline of the scrotal neck immediately after the rubber ring is applied. Two studies investigating the effect of this precise method of LA injection were identified during the literature search (Jongman et al., 2016; and Small et al., 2020) but were excluded as both studies were conducted in Australia and the lambs were over four weeks old. These studies do suggest that LA administered in this way has a limited impact on pain but there is currently no evidence regarding its use in lambs less than seven days old.

With the exception of one paper that used procaine 5% (Molony et al., 2012) all LA intervention used lignocaine 2%, however, lignocaine 2% is not available for use in food producing animals in the UK. While no local anaesthetics are licensed for sheep in the UK, procaine may be prescribed under the cascade. Small et al. (2021) suggests that procaine may have a similar onset time to lignocaine but that the effect may last longer in lambs castrated by rubber rings.

In conclusion, LA administered into the scrotal neck, spermatic cord and testes of lambs 15 minutes before castration with rubber rings may virtually eliminate pain. However, where this is not possible, injection of 0.3 ml of lignocaine 2% into the scrotal neck or each testis of a neonatal lamb at the time of castration may aid reduction of pain.

## Methodology

**Search strategy:** 

Databases searched and dates covered	CAB Abstracts on Cab Direct platform 1974–9 November 2023 Web of Science on Clarivate platform 1970– 9 November 2023
Search terms	CAB Abstracts: Castrat* (anaesthe* or anesthe* or lignocaine or lidocaine or procaine or mepivacaine or bupivacaine or analgesi* or numb or block) (lamb* or sheep or ovine or ovis) (pain or behaviour or behavior or welfare) 1 AND 2 AND 3 AND 4 Web of Science: castrat*(All Fields) anaesthe* (All Fields) or anesthe* (All Fields) or lignocaine (All Fields) or lidocaine (All Fields) or procaine(All Fields) or mepivacaine (All Fields) or bupivacaine (All Fields) or analgesi* (All Fields) or numb (All Fields) or block (All Fields) lamb*(All Fields) or sheep (All Fields) or ovine (All Fields) or ovis (All Fields) pain(All Fields) or behaviour (All Fields) or behavior (All Fields) or welfare (All Fields) #4 AND #3 AND #2 AND #1
Dates searches performed:	09 Nov 2023

**Exclusion / inclusion criteria:** 

Exclusion	Not relevant to PICO question, studies where castration was by surgery or Burdizzo, studies using species other than lambs, studies that did not use local anaesthetic as an intervention or did not have a group without local anaesthetic for comparison, studies that did not assess acute pain in response to castration, studies where the study population was older than 7 days, articles that are not primary studies, book chapters, narrative reviews and papers not available in English language.
Inclusion	Interventions included castration with rubber rings and castration with just local anaesthetic and castration with no anaesthetic.

**Search outcome:** 

Database	Number of results	Excluded – Not relevant to PICO question	Excluded – Lambs over 7 days old	Excluded – Surgical or Burdizzo castration	Excluded – Interventions did not include LA, or there was no control without LA	Excluded – Not primary studies	Excluded – Does not measure acute pain response	Excluded – Not English language or full text not available	Total relevant papers
CAB Abstracts	123	49	7	10	20	27	1	3	6
PubMed	196	148	9	11	7	16	0	1	4
Total relevant papers when duplicates removed									6

## ORCiD

Hannah Higgins: https://orcid.org/0000-0003-4416-1722

## Conflict of interest

The author declares no conflicts of interest.

## Disclaimer

Knowledge Summaries are a peer-reviewed article type which aims to answer a clinical question based on the best available current evidence. It does not override the responsibility of the practitioner. Informed decisions should be made by considering such factors as individual clinical expertise and judgement along with patient’s circumstances and owners’ values. Knowledge Summaries are a resource to help inform and any opinions expressed within the Knowledge Summaries are the author’s own and do not necessarily reflect the view of the RCVS Knowledge. Authors are responsible for the accuracy of the content. While the Editor and Publisher believe that all content herein are in accord with current recommendations and practice at the time of publication, they accept no legal responsibility for any errors or omissions, and make no warranty, express or implied, with respect to material contained within. For further information please refer to our Terms of Use.
